# FOXO1 regulates expression of a microRNA cluster on X chromosome

**DOI:** 10.18632/aging.100558

**Published:** 2013-05-12

**Authors:** Ruchi Singhal, Jonathan E. Bard, Norma J. Nowak, Michael J. Buck, Eugene S. Kandel

**Affiliations:** ^1^ Department of Cell Stress Biology, Roswell Park Cancer Institute, BLSC L3-318, Buffalo, NY 14263, USA; ^2^ Department of Biochemistry, Center of Excellence in Bioinformatics and Life Sciences, Buffalo NY 14203, USA

**Keywords:** the forkhead family of transcription factors, mir, miRNA, oncotarget, cancer

## Abstract

Phosphoinositol-3-kinase (PI3K) pathway is a crucial modulator of many physiological and pathophysiological phenomena, including aging, diabetes and cancer. Protein kinase Akt, a downstream effector of PI3K, controls a plethora of cellular functions, including gene transcription. A key mechanism connecting Akt activity to changes in gene expression is inhibitory phosphorylation of FOXO family of transcription factors. Accordingly, altered expression of FOXO targets may account for many biological consequences of PI3K/Akt signaling. While the previous efforts focused on FOXO-dependent regulation of protein-coding genes, non-coding RNA genes have emerged as equally important targets of many transcription factors. Therefore, we utilized a regulated form of FOXO1 to profile FOXO1-dependent changes in miRNA expression in human cells. Both microarray hybridization and next-generation sequencing revealed changes in the products of a miRNA cluster on X chromosome. Rapid induction of these miRNAs occurred independently of de novo protein synthesis. Furthermore, inhibition of PI3K in cancer cell lines caused derepression of these miRNAs, as would be expected for FOXO-regulated genes. Members of the major oncogenic cascades are significantly overrepresented among the predicted targets of the miRNAs, consistent with tumor-suppressive role of FOXO1. The discovered miRNAs represent new candidate mediators of FOXO1 functions and possible biomarkers of its activity.

## INTRODUCTION

Activation of the signal transduction cascade that proceeds through phosphoinisitol-3-phosphate kinase and protein kinase B (also known as AKT) is a frequent theme in signaling by various growth factors and a common subject of oncogenic alteration in cancer cells [1, 2]. A detailed knowledge of the consequences of activation of this pathway is essential for a better understanding of the function of a normal cell and may provide insights into the nature and possible mitigation of various disease states, including cancer, diabetes, and many others. Activation of AKT effects cellular changes through two broad groups of mechanisms. It modulates a large cohort of biochemical processes in a manner independent of de novo RNA synthesis. It also affects the levels of various cellular components through controlling the functions of transcription factors. Arguably, the best characterized AKT targets among the transcription factors are the proteins from the FOXO family [3]. AKT phosphorylates FOXO proteins on a series of highly conserved sites, leading to nuclear exclusion and inactivation of these molecules [4]. It is commonly accepted that many of the hallmark effects of AKT activation are directly attributable to FOXO inhibition, and that reactivation of FOXO influences cell response to therapies that target PI3K pathway [5]. This highlights the need to identify and investigate the targets of these transcription factors, especially in the view of the role of PI3K-AKT-FOXO connection in cancer, ageing and diabetes.

Traditionally, the focus of such research was on protein-coding genes, including the loci that control cell proliferation and survival. However, there is a growing appreciation of the role that non-protein coding RNAs play in the control of cellular processes. While some of these RNAs are direct components of signal transduction machinery [6], the best studied class are the so-called “micro RNAs” (miRNAs), which control translation of respective target mRNAs, and through that - a multitude of processes, such as cancer, senescence and differentiation [7-10]. In mammalian cells, typical miRNAs are first produced as imperfect hairpins within longer transcripts. Subsequent stepwise nucleolysis yields an imperfect duplex approximately 22 nucleotides in length, and one strand of this duplex is eventually incorporated into a translation silencing complex, where it serves as the determinant of specificity. Potentially, a single miRNA may target multiple mRNAs, which share with it short stretches of partial homology. There are also reports of miRNAs serving as components of multimolecular complexes that target gene expression at a promoter level (e.g.[11]).

It is increasingly clear that for most, if not all, of the mammalian transcription factors the understanding of the biological role remains incomplete until their effects on the miRNA expression are taken into account [12]. Furthermore, miRNAs are relatively well preserved in various biological samples and could be used as convenient reporters for the biological processes that regulate their expression (e.g. [13-15]). These considerations prompted us to search for the miRNAs, which are controlled by FOXO proteins in human cells.

## RESULTS

We have engineered a derivative of human embryonic kidney cell line HEK-293T for the expression of FOXO1-AAA-ER, a regulated form of human FOXO1 (also known as FKHR). The expressed protein contains T24A S256A S319A mutations, which make it insensitive to inhibition by AKT [4]. Furthermore, the protein is produced as a fusion with the ligand-binding domain of mouse estrogen receptor (ER). Therefore, the entire protein remains sequestered in the cytoplasm, until a suitable ligand is added. An additional mutation ensures that the ER fragment retains affinity to some artificial ligands (e.g. 4-hydroxitamoxifen or 4HT), but not to the natural estrogens [19]. Overall, the system provides tight regulation of FOXO1 activity under regular cell culture conditions in the presence of fetal bovine serum, which might contain Akt-activating factors or estrogen.

**Figure 1:**
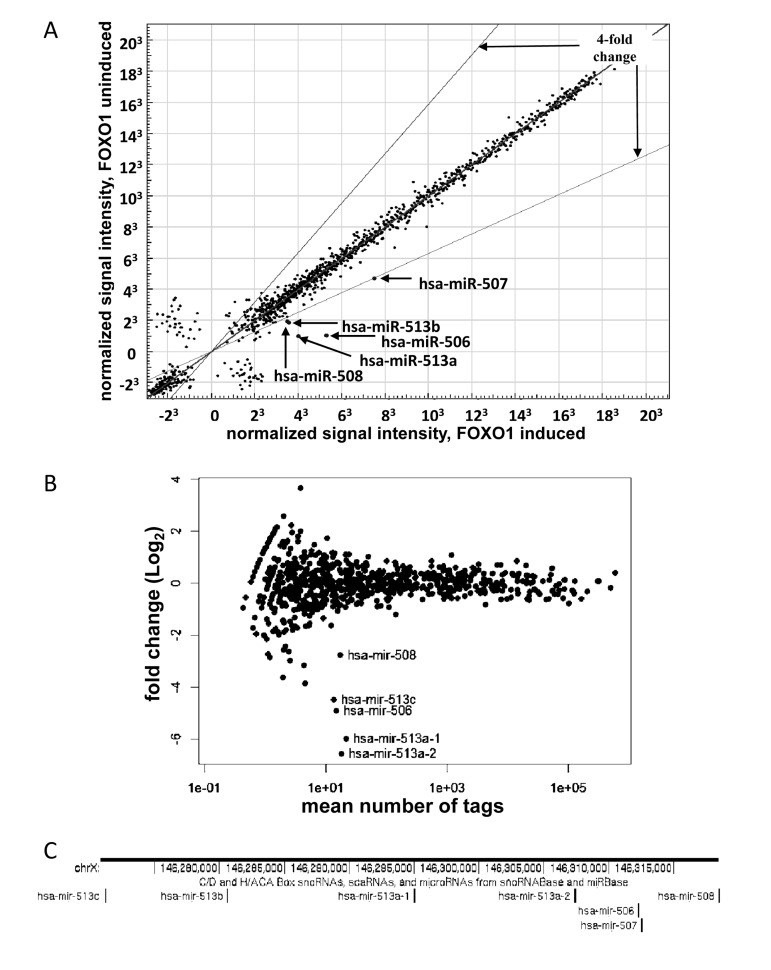
Activation of FOXO1-AAA-ER induces expression of a miRNA cluster on chromosome X. (**A**) Illumina Bead-Array comparison of expression profiles of miRNA from HEK-293T cells expressing FOXO-AAA-ER with or without an 8- hour treatment with 4-hydoxitamoxifen (inducer). The position of the indicated miRNAs on the blot is marked. The expression of the miRNAs outside of the indicated boundaries has changed by a factor of 4 or more. (**B**) Comparison of expression profiles of miRNA from HEK-293T cells expressing FOXO-AAA-ER with or without treatment with 4-hydoxitamixifen using Illumina next-generation sequencing. The position of the indicated miRNAs on the blot is marked. (**C**) Schematic representation of genomic localization of the differentially expressed miRNA genes. A fragment of X chromosome is shown annotated in UCSC Genome Browser (genome.ucsc.edu) with genomic coordinates and the track of small RNAs.

The miRNA pool from the FOXO1-AAA-ER -expressing cells, with or without 4HT treatment, was profiled using two alternative techniques: hybridization to miRNA-specific microarrays and high-throughput sequencing. Both methods yielded similar results (Fig 1A and 1B): a group of miRNAs, which originate from the same region of X chromosome (Figure 1C), were prominently induced upon activation of FOXO1. The miRNAs included miR-506, miR-507, miR-508, miR-513a-1, miR-513a-2 (highly homologous miR-513a-1 and miR-513a-2 were indistinguishable by array hybridization.), miR-513b and miR-513c. Fold induction could not be estimated for miR-513c in the microarray experiment: the signal was detectable upon induction, but was undetectable in uninduced control. For the same reason the fold induction of miR-507 could not been estimated in the high-throughput sequencing experiment. In these experiments, none of the miRNAs was induced by 4HT treatment in HEK-293T cells transduced with an empty vector in the absence of FOXO1-AAA-ER (data not shown).

**Figure 2:**
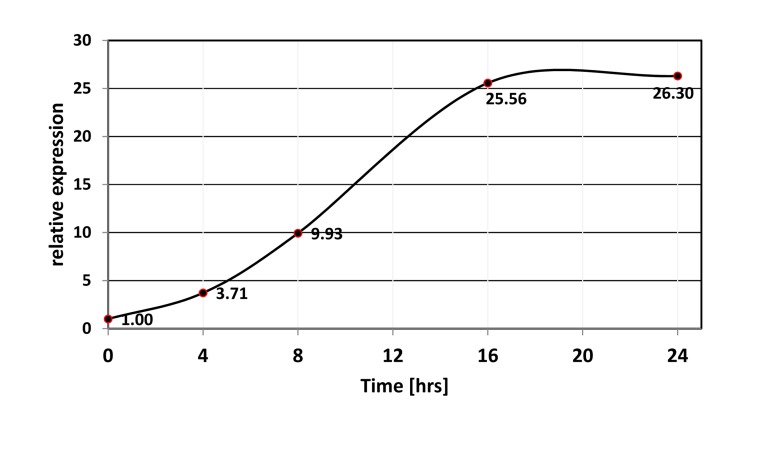
Time-course of miR-506 induction upon activation of FOXO1-AAA-ER. FOXO1-AAA-ER expressing HEK-293T cells were treated with 4-hydoxitamoxifen for the indicated periods of time, and the levels of miR-506 were compared by quantitative PCR using RNU6B as an internal control.

**Figure 3:**
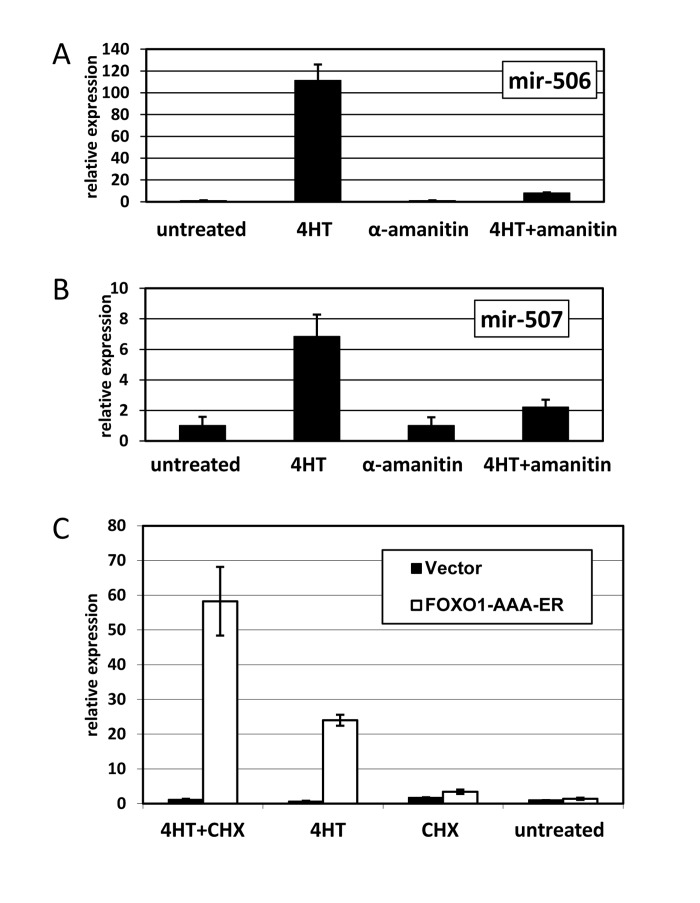
FOXO1 induction of miRNA expression depends on RNA polymerase II, but is independent of de novo protein synthesis. (**A-B**) Induction of miR-506 (**A**) and miR-507 (**B**) is prevented by an inhibitor of RNA polymerase II. HEK-293T cells expressing The cells were treated overnight as indicated. For combined treatment, α-amanitin was added 2 hours prior to addition of 4-hydoxitamoxifen (4HT). The levels of miR-506 (**A**) and miR-507 (**B**) were compared by quantitative PCR using RNU6B as an internal control and are shown relative to those in untreated cells. (**C**) Activation of FOXO1 induces miR-506 expression without de novo protein synthesis. HEK-293T cells harboring FOXO1-AAA-ER or the corresponding empty vector were treated for 8 hours with 4-hydoxitamoxifen (4HT), cycloheximide (protein synthesis inhibitor, CHX) or a combination thereof. The levels of miR-506 in treated and untreated cells were compared by quantitative PCR using RNU6B as an internal control and are shown relative to those of untreated vector-infected cells. An apparent increase in miR-506 levels in cycloheximide-treated vs. -untreated samples is due to a small decrease in RNU6B expression in the presence of cycloheximide (data not shown).

We used miR-506 as a representative member of the miRNA cluster. Quantitative RT-PCR analysis confirmed that activation of FOXO1 caused prominent induction of miR-506 in as little as 4 hours (Figure 2). The failure of 4HT to induce the changes in HEK-293 cells, which harbored an empty vector, confirmed that induction was dependent on the function of the expressed FOXO1 protein (Figure 3C).

Although there are reports of miRNA genes transcribed by RNA polymerase III [20], most miRNA appear to be transcribed by RNA polymerase II [21] and are regulated by RNA pol II-associated transcription factors, akin to protein-coding genes [22]. Induction of miR-506 and miR-507 expression was greatly attenuated by low doses of α-amanitin (Figure 3A and B), which selectively targets RNA pol II-dependent transcription. If FOXO1 is indeed a direct regulator of the miRNA transcription, induction should occur even in the absence of de novo protein synthesis. Accordingly, miR-506 was readily inducible in the presence of high doses of cycloheximide, a potent inhibitor of translation (Figure 3C). In fact, the apparent level of miR-506 was even higher in the presence of cycloheximide due to slightly decreased levels of the internal control (RNU6B) in these conditions. Overall, our data suggest that FOXO1 directly activates RNA polII-dependent transcription of the miRNA cluster.

**Figure 4:**
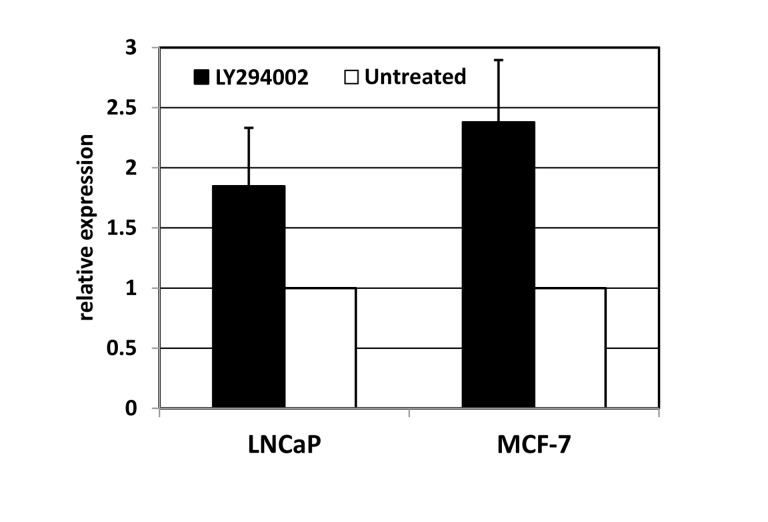
Inhibition of phosphoinositol-3-kinase elevates the expression of miRNA-506. LNCaP (prostate carcinoma) or MCF7 (breast carcinoma) cells were treated for 24 hours with PI3K inhibitor LY294002. The levels of miR-506 were measured using quantitative PCR with RNU6B as an internal standard, and normalized to those in untreated controls.

If the miRNAs are regulated by endogenous FOXO activity, one may expect their expression to be sensitive to the status of AKT in the same cells. In cancer cell lines, AKT activity is expected to be high, thus limiting activity of endogenous FOXO proteins. In order to verify whether expression of the miRNA cluster in cancer cells is suppressed by the PI3K/AKT axis, we used LY294002, a specific inhibitor of PI3K, in two human carcinoma cell lines: LNCaP and MCF7. In both cases, LY294002 treatment was accompanied by an increase in miR-506 expression, as measured by quantitative RT-PCR (Figure 4).

## DISCUSSION

Our findings demonstrate that expression of a cluster of miRNAs on chromosome X is susceptible to changes in the activity of FOXO1 transcription factor. The phenomenon depends on transcription by RNA polymerase II, but not de novo protein synthesis, suggesting that FOXO1 acts as a classical transcription factor and an immediate regulator of the miRNA expression. At present, the full structure of the corresponding transcripts is unknown. The miRNA may be generated from a single primary transcript or from multiple RNAs, each with its separate promoter. In our experiments we did not observe induction of miR-514b (data not shown), which is located approximately 10 kbps upstream of miR-508. Thus, it is tempting to speculate that at least one of the relevant promoters is located within that region. However, this does not necessarily mean that the relevant FOXO-binding sites have to be located within that area: there are ample examples of genes being affected by the DNA binding events at considerable distances [23, 24]. In fact, a recent study has indicated that FOXO3, which is functionally similar to FOXO1, may transactivate its target genes from distant enhancers via DNA looping [25].

Previously characterized FOXO targets include genes that encode growth suppressors, detoxifying enzymes, as well as regulators of metabolism [26], reflecting the broad range of biological roles of these transcription factors [27, 28]. Indeed, suppression of FOXO activity has been implicated in both tumor-promoting and cytotoxic effects of constitutive activation of PI3K/Akt pathway. The functions of the molecules from the miR-506 cluster are still unknown. One recent report has claimed that overexpressed miRNAs from this cluster, acting in concert with other miRNAs from the adjacent loci, contribute to cell survival and oncogenic transformation in melanoma [29], but no mechanistic explanation was given. So far, we were unable to see such an elevated expression of miR-506 in several melanoma cell lines (RS, ESK, unpublished). Also in melanoma, miRNAs from this cluster were found up-regulated in primary tumor cell lines in comparison to cultured melanocytes, yet downregulated, as invasive potential of melanoma cells increases [30]. There are further reports that downregulation of this cluster marks early relapse in advanced stage ovarian cancer patients [31] and expression of miR-506 inhibits NRAS expression and suppress growth and tumorigenesis in a lung cancer model [32]. Such a pattern of behavior would correspond to the cluster being a suppressor of oncogenic pathways and being negatively controlled by PI3K/Akt pathway, which is a key oncogenic force and is frequently upregulated in later-stage and metastatic cancers (e.g. [33]).

Interestingly, it was reported earlier that mouse homologue of FOXO3 binds DNA in the vicinity of a miRNA cluster consisting of miR-106b, miR-93 and miR-25; and the basal activity of this transcription factor may suppress the levels of these miRNA through an indirect mechanism [34, 35]. However, none of our profiling experiments revealed any appreciable changes in these three miRNA (data not shown). The discrepancy may reflect the difference between FOXO family members or between the cells used in these experiments (e.g. human vs. mouse origin).

It is important to note that bioinformatic prediction of individual miRNA targets is not very accurate[36]. However, a single miRNA is likely to regulate a biological process by targeting more than one involved gene, and co-expressed clusters of miRNAs have been reported to cooperatively control the members of the same biochemical pathway or even of the same protein complex [37]. Hence, the biological function of a miRNA cluster may be implied from the list of pathways, whose components are significantly overrepresented among the predicted miRNA targets. To this end, we used the tools at DIANA LAB web site (http://diana.cslab.ece.ntua.gr/) [38] to identify the pathways from the Kyoto Encyclopedia of Genes and Genomes (KeGG), which are significantly overrepresented among the targets of the miRNA cluster (Supplementary Table S1). Targets were predicted using TargetScan [39], and their list may imply a tumor-suppressive function for the miRNAs. On the list are some anti-apoptotic factors (NFKB1, BCL2L1, etc.). Prominently featured are the activators and the members of the canonical PI3K pathway (IGF1, IGF1R, PDFGRA, IRS2, PIK3CA, PIK3R3, AKT2, etc.). Also abundant among the predicted targets are various activators, intermediaries and downstream effectors of the MAP kinase cascade (e.g. KRAS, RRAS, SOS1, SOS2, MAP3K1, MAP3K3, MAP3K4, MAP2K7, MAPK1, MAPK7, MAPK14, ETS1, ETS2 and many more). PAK1, which acts as a liaison between the PI3K and MAPK pathways [40, 41], is on the list as well. Not surprisingly, the biological processes where these biochemical pathways play prominent roles are predicted to be affected by the miRNA cluster (Supplementary Table S1). These include the geneses of multiple types of cancers; regulation of cell adhesion, migration and cytoskeleton organization; signaling by various cytokines and growth factors; and some of the metabolic and immune functions. In general, this is consistent with the known biological consequences of activation of FOXO factors in mammalian cells [42]. Interestingly, FOXO1 and FOXO3 are also on the list of predicted targets, suggesting a possibility of a feedback loop that may control the abundance of these proteins. Such transcription factor - miRNA circuits appear to be a general theme in the control of mammalian gene expression [12].

Our findings point to a previously unknown facet of the PI3K-Akt-FOXO signaling. Considering the widely acknowledged involvement of this pathway in cancer, diabetes and many other pathological conditions [42], a further study of the identified miRNAs is likely to yield insights into the molecular underpinning and possible biomarkers of these phenomena.

## MATERIALS AND METHODS

### Cell culture

All cells were cultured in humidified chambers at 37°C and 5% CO2 in high-glucose DMEM supplemented with L-glutamine (4 mM), fetal bovine serum (10%), penicillin (100 U/ml) and streptomycin (100 μg/ml). Cells were free of mycoplasma contamination, as tested using MycoAlert Mycoplasma Detection Kit (Lonza). 4-hydroxitamoxifen, α-amanitin, cycloheximide and LY294002 were used at 1μM, 0.4 μg/ml, 10μg/ml and 60μM respectively.

### Retroviral transduction

FOXO1-AAA-ER was expressed from pLPCX retroviral vector (Clontech, Inc.). Recombinant retroviral stocks were produced by co-transfecting 293T cells with an appropriate vector and a packaging construct (pCL10A1 from Imgenex, Inc). Transfections were carried out using Lipofectamine Plus (Life Technologies Corporation) according to the manufacturer's recommendations. Viral stocks were applied as previously described [16], and the infected cells were selected on puromycin (1μg/ml).

### miRNA profiling

For miRNA profiling, HEK-293 cells transduced with either FOXO1-AAA-ER or an empty vector were plated on two 10 cm plates, 4.0×10^6^ cells per plate. For each cell line, one plate of cells was treated with 1 μM 4HT for 8 hours while the other was kept as control (untreated). RNA was then extracted using TRIzol Reagent (Invitrogen, Inc.) according to the manufacturer's protocol.

Microarray hybridization was performed at the Genomics Facility of the Roswell Park Cancer Institute using miRNA-specific BeadArray (Illumina) according to the manufacturer's specification and analyzed using Illumina GenomeStudio software.

The same samples were also prepared for Illumina next generation sequencing using TruSeq Small RNA protocol (Illumina) with library size selection performed on a Pippin Prep (Sage Science). Sequencing was done on Illumina HiSeq machine. Illumina sequencing reads were demultiplexed with CASAVA 1.8, trimmed for adaptor sequence, and aligned to the human genome with BowTie [17]. Aligned reads at miRBase (r18) annotated miRNAs were then tabulated. Differential expressed miRNA (highlighted in red) were determined using the DESeq R package [18] with parametric normalization at FDR < 10%.

### Quantitative RT-PCR

RNA was isolated using mirVana miRNA Isoloation kit (Ambion) and expression of mature miRNA was measured by TaqMan miRNA assays (Applied Biosystems). RNU6B was used an internal control. Quantitative PCR was done using miRNA- or RNU6B-specific primers on triplicate samples in a 7900HT thermocycler (Applied Biosystems). The denaturation step at 95°C was done for 15 seconds, and the annealing and extension step was done at 60°C for 1 min. SDS 2.3 software (Applied Biosystems) was used to identify cycle threshold (Ct) values. The data were normalized to RNU6B expression.

## Supplementary Tables


