# Supplementary material for: FOXO1 regulates expression of a microRNA cluster on X chromosome
Source: Aging (Albany NY). 2013 May 12;5(5):347–56. doi: 10.18632/aging.100558 (PMC3701110; doi:10.18632/aging.100558)
Supplement: Supplementary file 1 [file aging-05-347-s001.pdf]

Supplementary Table S1. Predicted pathways and genes targeted by the FOXO1-responsive miRNAs.

KEGG Pathway	Pathway ID	all miR combined	miR-506	miR-507	miR-508	miR-513a	miR-513b	miR-513c	Predicted individual targets (all miRNA combined)
Axon guidance	hsa04360								EFNB2, SRGAP3, SEMA6A, EPHA3, NCK2, GNAI2, SLIT1, ABLIM3, CXCL12, ITGB1, SEMA6C, PLXNB2, LRRC4C, MET, GNAI3, SEMA6D, SRGAP2, ROCK1, KRAS, SEMA6B, NRP1, PAK1, MAPK1, NFATC1, PAK6, SEMA4G, PPP3CA, EPHA8, PLXNA3, SEMA5A, EFNB3, NFAT5, EFNB1
Colorectal cancer	hsa05210								PIK3CA, RALGDS, AXIN1, LEF1, DVL3, PDGFRA, FZD5, TGFBR1, IGF1R, MET, SOS2, FZD8, KRAS, SMAD2, CYCS, FZD3, SOS1, APC, MAPK1, GRB2, AKT2, FZD4, PIK3R3, SMAD4
Chronic myeloid leukemia	hsa05220								PIK3CA, E2F3, EVI1, TGFBR1, NFKB1, SOS2, KRAS, STAT5B, CBL, SOS1, PTPN11, SHC1, MAPK1, RUNX1, GRB2, AKT2, SHC4, PIK3R3, BCL2L1, CTBP2, SMAD4
TGF-beta signaling pathway	hsa04350								E2F5, TGFBR1, DOCK5, ID2, BMP6, ROCK1, SMURF1, SMAD7, SMAD2, RBL1, SMAD5, ACVR2A, GDF6, MAPK1, PPP2R2C, THBS2, PPP2CA, ACVR2B, BMPR1B, SP1, BMP4, RPS6KB1, SMAD4
Dorso-ventral axis formation	hsa04320								NOTCH2, SPIRE1, SOS2, KRAS, SOS1, MAPK1, GRB2, ETS2, ERBB4, ETS1
ErbB signaling pathway	hsa04012								CAMK2D, NCK2, PIK3CA, SOS2, KRAS, STAT5B, CBL, MAP2K4, SOS1, PAK1, SHC1, MAPK1, SRC, ABL2, GRB2, PAK6, MAP2K7, AKT2, ERBB4, SHC4, RPS6KB1, PIK3R3
Insulin signaling pathway	hsa04910								IRS2, PRKAG2, PIK3CA, PTPN1, PPP1R3B, TSC1, LIPE, SOS2, KRAS, CBL, GYS1, SREBF1, PRKAA2, FLOT2, SOS1, SOCS2, FOXO1, SHC1, MAPK1, PHKB, GRB2, SOCS3, FLOT1, AKT2, PHKA1, SHC4, RPS6KB1, PIK3R3, PPP1R3D
Acute myeloid leukemia	hsa05221								PIK3CA, LEF1, STAT3, NFKB1, PIM1, SOS2, KRAS, PML, SOS1, MAPK1, RUNX1, GRB2, AKT2, RPS6KB1, PIK3R3
Adherens junction	hsa04520								PTPN1, LEF1, TGFBR1, IGF1R, MET, WASF3, WASL, SMAD2, CTNNA2, MAPK1, PTPRJ, SRC, PVRL2, SSX2IP, ACTN4, IQGAP1, SMAD4, SNAI2
Benzoate degradation via CoA ligation	hsa00632								SH3GLB1, DHRS1, MYST3, ESCO1, YOD1, GCDH, LYCAT, HADHA, MYST4
mTOR signaling pathway	hsa04150								PIK3CA, RPS6KA1, PGF, TSC1, FIGF, PRKAA2, MAPK1, ULK2, AKT2, RPS6KA3, IGF1, RPS6KB1, PIK3R3
Epithelial cell signaling in Helicobacter pylori	hsa05120								PTPRZ1, ATP6V1C1, NFKB1, MET, ATP6V0E1, MAP2K4, ATP6V1C2, PTPN11, PAK1, SRC, ADAM17, ATP6V1A, ATP6V1B2, ATP6V0A2, MAPK14, ATP6V0D2
Focal adhesion	hsa04510								ITGA3, COL6A3, PIK3CA, VASP, PGF, ITGB1, PDGFRA, IGF1R, CAPN2, MET, DIAPH1, FIGF, ROCK1, SOS2, ITGB8, SOS1, PAK1, SHC1, MAPK1, TLN1, SRC, GRB2, PAK6, THBS2, CAV1, ITGB3, AKT2, ITGAV, ACTN4, CCND2, LAMC1, IGF1, ITGA7, SHC4, PIK3R3, COL4A1
Regulation of actin cytoskeleton	hsa04810								ITGA3, MYH9, PIK3CA, ITGB1, PDGFRA, IQGAP2, DIAPH1, SSH2, CYFIP2, ROCK1, PPP1R12B, WASL, SOS2, ITGB8, KRAS, ARPC1B, SOS1, FGF9, ARHGEF1, PAK1, APC, MAPK1, FGF7, DIAPH2, ARHGEF4, PIP4K2C, PAK6, ITGB3, ITGAV, ACTN4, GSN, PIP5K3, MYH10, RRAS, ITGA7, PIK3R3, IQGAP1, PFN2
Ubiquitin mediated proteolysis	hsa04120								UBE2G2, UBE2G1, UBE4A, UBE3A, UBE2NL, UBE2R2, MAP3K1, CUL3, SMURF1, WWP2, UBE2W, CBL, MID1, PML, UBE2B, UBE2L3, UBE2N, FBXW7, WWP1, FBXW11, TRIP12, SOCS3, PIAS3, UBE2O, CUL5, UBE2H
Wnt signaling pathway	hsa04310								CAMK2D, DAAM2, AXIN1, LEF1, DVL3, FZD5, DOCK5, LRP6, CSNK1A1L, ROCK1, FZD8, SMAD2, FZD3, APC, VANGL1, PPP2R2C, FBXW11, NFATC1, PPP2CA, PPP3CA, PLCB1, CCND2, SFRP5, FZD4, NFAT5, PRICKLE2, CTBP2, SMAD4
Antigen processing and presentation	hsa04612								NFYB

Glioma	hsa05214								CAMK2D, PIK3CA, E2F3, PDGFRA, IGF1R, SOS2, KRAS, SOS1, SHC1, MAPK1, GRB2, AKT2, IGF1, SHC4, PIK3R3
Endometrial cancer	hsa05213								PIK3CA, AXIN1, LEF1, FOXO3, SOS2, KRAS, SOS1, CTNNA2, APC, MAPK1, GRB2, AKT2, PIK3R3
Tight junction	hsa04530								MYH9, GNAI2, TJP2, PRKCQ, DOCK5, GNAI3, VAPA, KRAS, PRKCE, PPP2R3A, CLDN11, CTNNA2, EXOC4, ASH1L, PPP2R2C, AMOTL1, SRC, MYH11, PPP2CA, PARD6B, AKT2, CGN, ACTN4, TJAP1, MYH10, RRAS
Cytokine-cytokine receptor interaction	hsa04060								IL2RG, TNFSF4, CXCL12, TNFSF15, PDGFRA, EDA, TGFBR1, MET, LIFR, CXCL5, ACVR2A, GHR, ACVR2B, TNFRSF19, BMPR1B, IL11, IL6R
Complement and coagulation cascades	hsa04610								CD55
Non-small cell lung cancer	hsa05223								PIK3CA, RASSF5, E2F3, FOXO3, SOS2, KRAS, STK4, SOS1, MAPK1, GRB2, AKT2, PIK3R3, RXRA
Pancreatic cancer	hsa05212								PIK3CA, RALGDS, RALBP1, E2F3, PGF, STAT3, TGFBR1, NFKB1, FIGF, KRAS, SMAD2, MAPK1, AKT2, PIK3R3, BCL2L1, SMAD4
Glycolysis / Gluconeogenesis	hsa00010								PGM1
Cell Communication	hsa01430								COL6A3, DSC2, VIM, KRT12, THBS2, LAMC1, COL4A1
Limonene and pinene degradation	hsa00903								SH3GLB1, DHRS1, MYST3, ESCO1, YOD1, LYCAT, HADHA, MYST4
Renal cell carcinoma	hsa05211								PIK3CA, PGF, MET, FIGF, SOS2, KRAS, SOS1, PTPN11, PAK1, MAPK1, GRB2, PAK6, AKT2, ETS1, PIK3R3
Metabolism of xenobiotics by cytochrome P450	hsa00980								GSTK1
Polyunsaturated fatty acid biosynthesis	hsa01040								SCD, ACOX1, ELOVL5, YOD1, PECR, HADHA
Oxidative phosphorylation	hsa00190								ATP6V1C1, ATP6V0E1, ATP6V1C2, ATP6V1A, ATP6V1B2, ATP6V0A2, ATP6V0D2
T cell receptor signaling pathway	hsa04660								NCK2, PIK3CA, PRKCQ, NFKB1, SOS2, KRAS, CBL, SOS1, PAK1, NFATC1, GRB2, PAK6, PPP3CA, AKT2, NFAT5, CD28, PIK3R3
Adipocytokine signaling pathway	hsa04920								IRS2, PRKAG2, STAT3, PRKCQ, ACSL1, NFKB1, PRKAA2, ACSL3, ADIPOR2, PTPN11, SOCS3, AKT2, RXRA, PPARA
Glycan structures - biosynthesis 1	hsa01030								NDST1, CHST14, ST3GAL3, GALNT13, C1GALT1, MAN1A1, HS6ST3, ALG9, CHST1, GALNT9, GALNT10, MGAT4A, GCNT1, EXTL2, B4GALT5, EXTL3, GALNT12, ALG2, CHSY1, B4GALT1, XYLT1
Small cell lung cancer	hsa05222								ITGA3, TRAF4, PIK3CA, E2F3, ITGB1, NFKB1, CYCS, PTGS2, AKT2, ITGAV, PIAS3, LAMC1, PIK3R3, BCL2L1, COL4A1, RXRA
GnRH signaling pathway	hsa04912								CAMK2D, ADCY1, MAP3K1, SOS2, KRAS, MAP2K4, SOS1, ITPR3, MAP3K3, MAPK1, SRC, MAPK7, GRB2, ADCY9, MAP2K7, MAPK14, PLCB1
Heparan sulfate biosynthesis	hsa00534								NDST1, HS6ST3, GLCE, EXTL2, EXTL3
Dentatorubropallidoluysian atrophy (DRPLA)	hsa05050								WWP2, CASP7, WWP1, RERE
Melanogenesis	hsa04916								CAMK2D, GNAI2, LEF1, DVL3, FZD5, EDNRB, GNAI3, ADCY1, FZD8, KRAS, FZD3, MITF, MAPK1, ADCY9, PLCB1, FZD4, CREB3L2
Fatty acid elongation in mitochondria	hsa00062								HADH, ACAA2, HADHA
Parkinson's disease	hsa05020								UBE2G2, UBE2G1, SNCA, UBE2L3, STX1A

Melanoma	hsa05218								PIK3CA, E2F3, PDGFRA, IGF1R, MET, KRAS, FGF9, MITF, MAPK1, FGF7, AKT2, IGF1, PIK3R3
Cell adhesion molecules (CAMs)	hsa04514								MPZL1, NRCAM, ITGB1, CDH2, VCAN, SDC4, NEGR1, ITGB8, CNTNAP2, NFASC, NEO1, CLDN11, SPN, ALCAM, PVRL2, CADM1, ITGAV, CNTN1, CD28, NRXN3
Basal cell carcinoma	hsa05217								AXIN1, LEF1, DVL3, FZD5, GLI3, FZD8, FZD3, APC, FZD4, BMP4
Notch signaling pathway	hsa04330								NUMB, DVL3, NOTCH2, DLL1, ADAM17, JAG1, JAG2, CTBP2
VEGF signaling pathway	hsa04370								PIK3CA, KRAS, SPHK1, PTGS2, MAPK1, SRC, NFATC1, PPP3CA, AKT2, MAPK14, NFAT5, PIK3R3
Gap junction	hsa04540								GNAI2, PDGFRA, GNAI3, ADCY1, SOS2, KRAS, HTR2C, SOS1, ITPR3, MAPK1, SRC, MAPK7, GRB2, ADCY9, PLCB1
Cholera - Infection	hsa05110								ATP6V1C1, ATP6V0E1, ATP6V1C2, ATP6V1A, ADCY9, ATP6V0A2, ATP6V0D2
MAPK signaling pathway	hsa04010								RPS6KA1, RASGRP2, PDGFRA, EVI1, TGFBR1, NFKB1, MAP3K1, SOS2, KRAS, CACNB2, BDNF, NR4A1, STK4, MAP2K4, RPS6KA4, SOS1, FGF9, MAP3K3, PAK1, MAPK1, MAPK7, FGF7, GRB2, MAP2K7, PPP3CA, AKT2, DUSP3, MAPK14, PTPRR, RPS6KA3, RRAS, MAP2K1IP1, CACNB3
Glutamate metabolism	hsa00251								GFPT1, GPT2, GLS, GFPT2, CPS1
Neurodegenerative Diseases	hsa01510								VAPB, SNCA, CASP7, FBXW7, GRB2, BCL2L1
Maturity onset diabetes of the young	hsa04950								NEUROD1, PAX6, NKX2-2, HNF4G
Ether lipid metabolism	hsa00565								AGPAT3, ENPP6, LYCAT, PAFAH1B1
Pathogenic Escherichia coli infection - EHEC	hsa05130								NCK2, ITGB1, ROCK1, WASL, YWHAQ, YWHAZ
Pathogenic Escherichia coli infection - EPEC	hsa05131								NCK2, ITGB1, ROCK1, WASL, YWHAQ, YWHAZ

The miRNA targets were predicted using TargetScan-5 and assigned to KEGG pathways using the tools at DIANA LAB web site (http://diana.cslab.ece.ntua.gr/). The table includes the pathways that were significantly overrepresented among the predicted targets of the indicated miRNAs, as well as the individual members of those pathways, which are predicted targets of one or more miRNAs. The cells are color-coded according to the statistical significance of enrichment of a given pathway among the targets of the indicated miRNA:

$p<0.01$

$0.01<p<0.05$

$0.05<p<0.1$

$p>0.1$

no genes from the pathway
